# Anatomy and histology of the olfactory organ of Korean amur goby *Rhinogobius brunneus* (Gobiiformes, Gobiidae)

**DOI:** 10.1186/s42649-025-00116-4

**Published:** 2025-11-29

**Authors:** Hyun Tae Kim

**Affiliations:** Department of Science Education, Jeonju National University of Education, Jeonju, 55101 Republic of Korea

**Keywords:** Tubular nostril, Single lamella, Two accessory sacs, Mucous cell, Glycoprotein

## Abstract

This study investigated the anatomy and histology of the olfactory organ of the Korean amur goby *Rhinogobius brunneus* from Jeonjucheon stream. This species lives in shallow, stagnant, and intermittently low-oxygenated streams, reservoirs, and ponds affected by seasonal rainfall. Anatomically, its olfactory organ consisted of a short tubular anterior nostril, a posterior nostril, a single longitudinal lamella, and two accessory nasal sacs (ethmoidal and lacrymal sacs). Its single lamella structure resembles other gobiid fishes with a simplified olfactory surface. Histologically, the sensory epithelium comprised olfactory receptor neurons, supporting cells, and basal cells, while the non-sensory epithelium contained stratified epithelial cells, basal cells, and mucous cells. In particular, the mucous cells stained intensely red with Hematoxylin and eosin and Masson’s trichrome, indicating proteinaceous granular mucins rich in glycoproteins; such secretions likely protect the epithelium against particles, toxins, and low-oxygenated freshwater. Overall, the goby’s olfactory organ exhibits traits uniquely adapted to stagnant and hypoxic conditions.

**Supplementary Information:**

The online version contains supplementary material available at 10.1186/s42649-025-00116-4.

## Introduction

Teleost fishes with extensive adaptive radiation inhabit diverse aquatic habitats, and possess sensory systems highly specialized to their ecological niches (Escobar-Camacho and Carleton 2015). Among these senses, the olfactory organ plays a crucial role in mediating ecologically significant behaviors, such as foraging, predator avoidance, mate recognition, migration, and habitat selection (Hara 1986). These ecologies with physical characteristics of the habitat continuously influence the morphological peculiarity and histological modification in the olfactory organ of fishes (Kasumyan 2004). Therefore, the olfactory organ, including external nostril, olfactory epithelium, nasal sac, and the olfactory bulb, exhibits remarkable structural diversity across species (Zeiske et al. 1992). In particular, adaptation to aquatic environments with low oxygen levels or minimal water availability has led gobiid species to exhibit well-developed mucous cells within the olfactory epithelium and abundant blood capillaries within the dermis (Ghosh 2020; Kim 2024).

The Korean amur goby *Rhinogobius brunneus* of family Gobiidae is distributed throughout Japan, Taiwan, China, The Philippines, and the Korean Peninsula, and typically inhabits the middle to upper reaches of rivers, preferring bottom areas with gravel or rocky substrates (Kim and Park 2002). The goby is an omnivorous fish that feeds on aquatic insects, invertebrates, and juvenile fish, and males exhibit parental care by guarding the eggs beneath stones during the breeding season (Suk and Choe 2002). In South Korea, its habitat is a lotic freshwater system that routinely encounters extreme low-water levels with low oxygen saturation and seasonally shallow, slow-moving, and stagnant water (Jo et al. 2023). To cope with these adverse environmental conditions, some gobies exhibit histological adaptation, such as the presence of abundant capillaries within the epidermis and dermis that facilitate cutaneous respiration (Kurita et al. 2021; Kim et al. 2022). In this regard, the olfactory organs of *R. brunneus* are expected to exhibit adaptive characteristics similar to those observed in its skin. The morphology and anatomical and histological characteristics of bony fishes can provide important insight into their adaptations to habitat and life history, taxonomic and evolutionary relationships among species, and ecological roles such as feeding, respiration, and reproduction (Baxter et al. 2022). However, histological studies relating *R. brunneus* habitat and ecology to its anatomical and histological features have so far been conducted only on the skin. This study aimed to elucidate the relationship of the habitat and ecology of this Korean goby with the anatomical and histological characteristics of its olfactory organs.

## Materials and methods

### Specimen collection and fixation

**Fig. 1:**
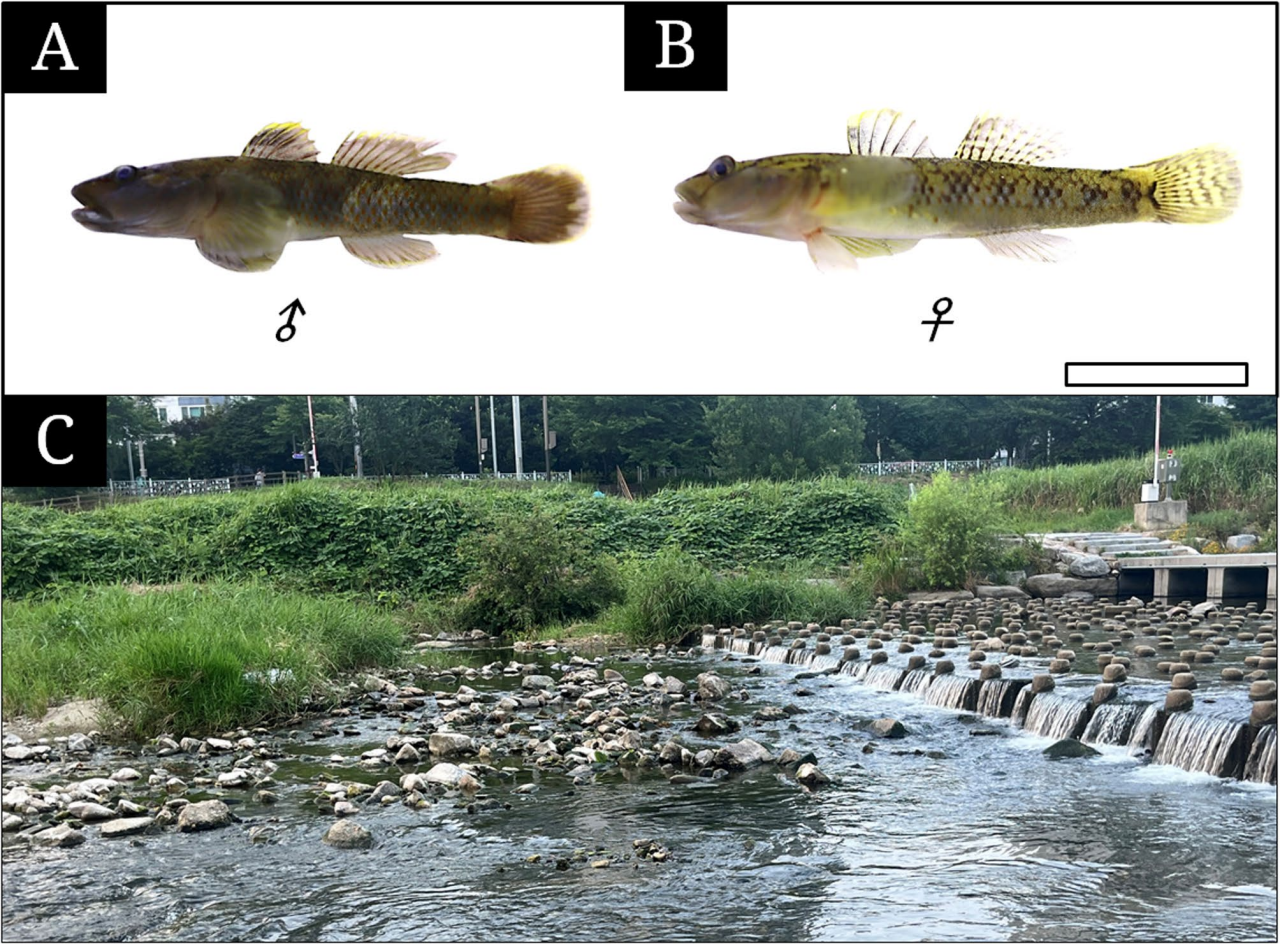
The photograph (**A**, male; **B**, female) of Rhinogobius brunneus and its habitat (**C**). The bar indicates 2 cm

In June to July 2025, 10 adult *R. brunneus* (standard length: 42–71 mm; Fig. 1 A and B) were collected from the middle reaches of Jeonjucheon stream at Deokjin-dong, Deokjin-gu, Jeonju-si, Jeonlabuk-do, South Korea (35°50’18”N, 127°06’41”E, Fig. 1 C) using a 4 × 4 mm mesh scoop net. Specimens were transported to the laboratory, anesthetized with MS-222 (Sigma, St. Louis, MO, USA), and fixed either in 10% neutral buffered formalin (pH 7.4; *n* = 6) for general histology or 2.5% glutaraldehyde (GA) buffer (pH 7.4; *n* = 4). All procedures complied with the guidelines of the Jeonbuk National University Institutional Animal Care and Use Committee.

## Microscopy procedures

The olfactory tissues of the goby were carefully excised using a surgical blade and subsequently documented with a stereomicroscope (Stemi DV4, Carl Zeiss, Jena, Germany) coupled to a digital camera (TG-3, Olympus, Japan). For light microscopic analysis, specimens preserved in formalin were rinsed under running tap water for 24 h, followed by dehydration through a graded ethanol series (70%, 80%, 90%, 95%, and then 100%). The tissues were then cleared in xylene, embedded in paraffin, and sectioned serially at a thickness of 5 μm. Sections were stained with hematoxylin and eosin (H&E) and Masson’s trichrome, and examined using a light microscope (Axio Imager. A2, Carl Zeiss, Oberkochen, Germany). For scanning electron microscopy, tissues fixed in glutaraldehyde (GA) solution were washed in 0.1 M potassium phosphate buffer (pH 7.4) for three cycles of 20 min each, post-fixed in 2% osmium tetroxide (OsO₄) buffer solution (pH 7.4), dehydrated through a graded ethanol series (50–100%), and immersed in tert-butyl alcohol. Samples were freeze-dried (VFD-21 S, Vacuum Device Co., Ltd., Ibaragi, Japan), coated with OsO₄, and observed under a scanning electron microscope (SUPRA40VP, Carl Zeiss, Germany).

## Results

### Anatomy

**Fig. 2:**
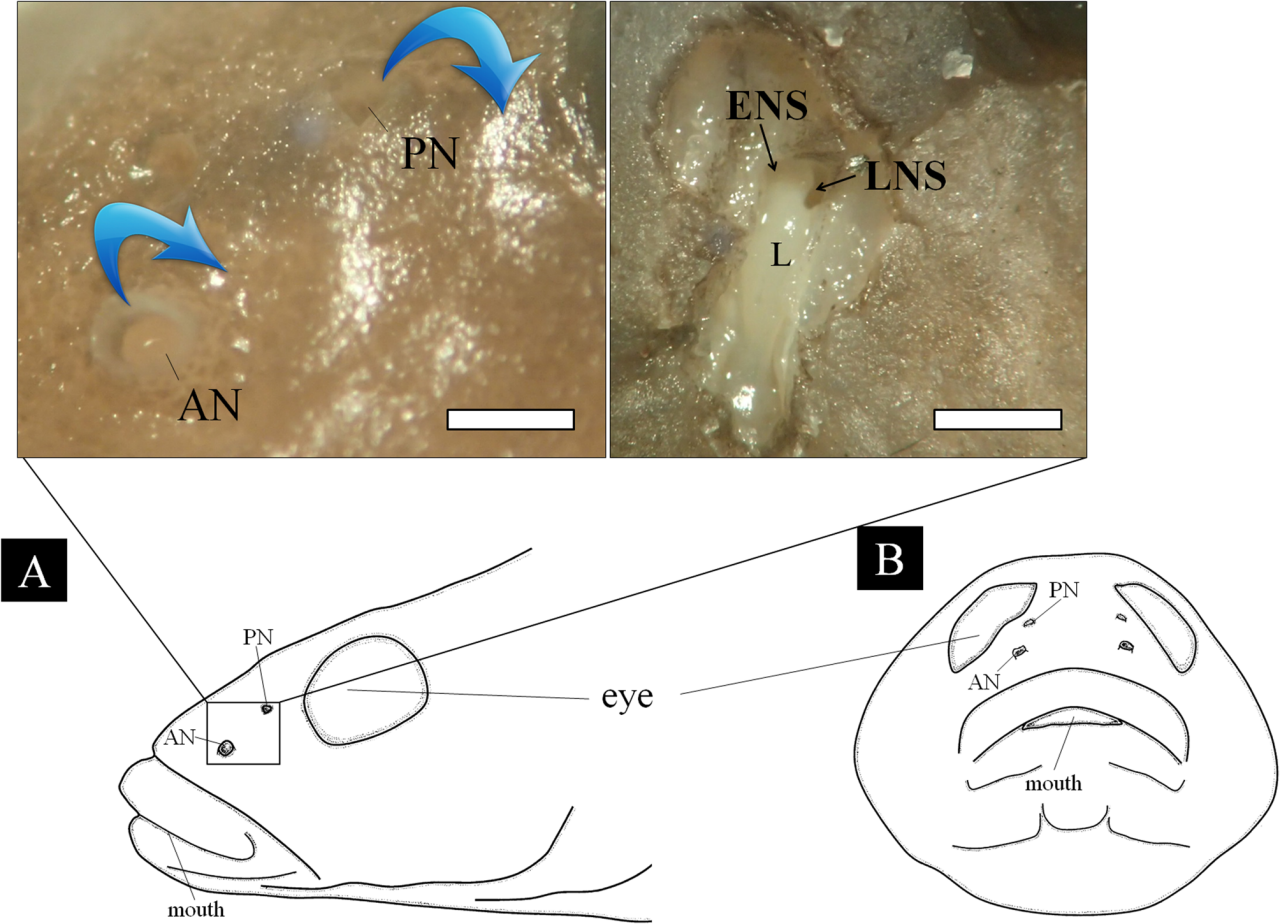
Schematic diagram of the lateral (**A**) and front (**B**) view of the head of Rhinogobius brunneus and the external (left photograph) and internal (right) structure of its olfactory organ. The blue arrow indicates water flowing. AN, anterior nostril; ENS, ethmoidal nasal sac; L, lamellae; LNS, lacrymal nasal sac; PN, posterior nostril

The paired olfactory organs of *R. brunneus* are positioned dorsally on the snout (Fig. 2 A and B). Each organ opens to the exterior through two apertures, the anterior nostril (AN) and posterior nostril (PN), which are separated by a distance of 0.74–1.23 mm. The AN forms a short tubular structure measuring 0.34–0.48 mm in diameter, whereas the PN, smaller in size, 0.18–0.35 mm in diameter, is flush with the surface rather than protruding. Within the olfactory chamber, a single longitudinally-folded lamella is present posteriorly with two accessory nasal sacs, the ethmoidal (ENS) and lacrimal sacs (LNS). The ENS lies below the posterior nostril and extends medially between the orbits, whereas the LNS is located in the posterior part of the olfactory chamber (OC) and extends ventrally into the suborbital region.

## Histology

**Fig. 3:**
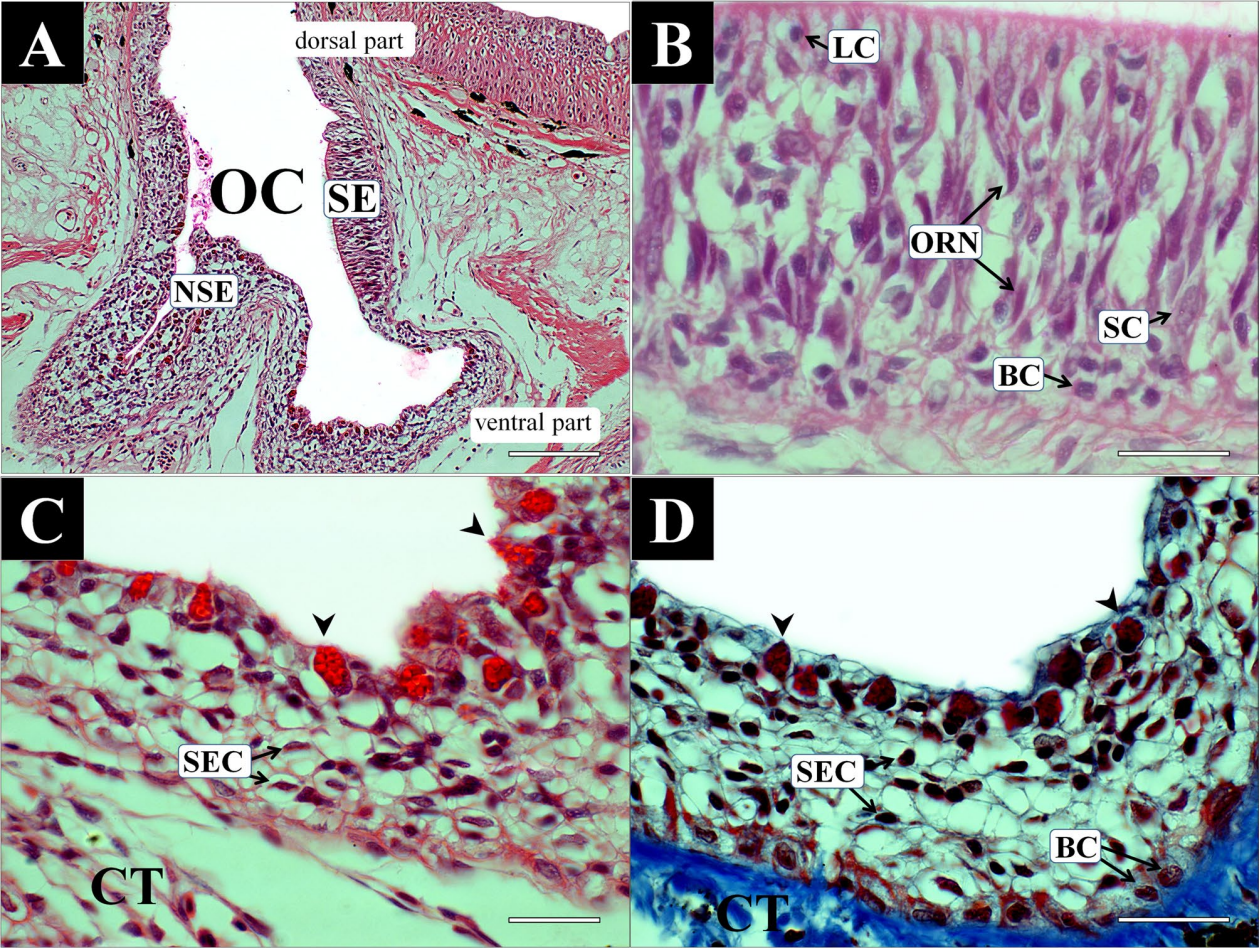
Histological characteristics of the olfactory epithelia of Rhinogobius brunneus, stained with hematoxylin and eosin (**A**, **B**, **C**), Masson’s trichrome (**D**). **A**, the olfactory chamber consisting of sensory epithelium and non-sensory epithelium; **B**, the sensory epithelium with olfactory receptor neurons, supporting cells, basal cells, and lymphatic cells; **C** and **D**, the non-sensory epithelium containing stratified epithelial cells, basal cells, and mucous cells. Arrowheads indicate mucous cells. BC, basal cell; CT, connective tissue; LC, lymphatic cell; NSE, non-sensory epithelium; OC, olfactory chamber; ORN, olfactory receptor neuron; SE, sensory epithelium; SEC, stratified epithelial cell; SC, supporting cells. The bars indicate 200 μm in **A** and 50 μm in **B**, **C** and **D**, respectively

On the cross-sectional view, the olfactory chamber contained the sensory epithelia (SE) and non-sensory epithelia (NSE) (Fig. 3 A). The SE comprised several cell types, including olfactory receptor neurons (ORN), supporting cells (SC), and basal cells (BC), and lymphatic cells (LC) (Fig. 3 B). ORNs were bipolar neurons characterized by an elongated nucleus and cytoplasm extending from the basement membrane to the epithelial surface. On H&E staining, their nuclei appeared purple, whereas the cytoplasm was faintly pink. SCs were cylindrical cells extending from the basal layer to the surface, each containing an oval nucleus weakly stained in purple and broad cytoplasm under H&E. BCs were rounded cells located just above the basement membrane, distinguished by a darkly stained purple nucleus. LCs were the smallest circular cells, intensely violet in color, and distributed mainly in the basal and upper epithelial layers.

The NSE was composed of stratified epithelial cells (SEC), mucous cells (MC), and BCs (Fig. 3 C and D). SECs were polygonal in shape, with round or flattened nuclei and broad cytoplasm that showed little to no affinity for either H&E or Masson’s trichrome stains. These cells formed the predominant component of the epithelial layer. MCs were goblet-shaped cells resembling a grape cluster, and contained conspicuous mucous granules that stained bright red with H&E and deep red with Masson’s trichrome. Their nuclei were flattened and located basally. BCs within the NSE appeared as large round cells with dark brownish nuclei under Masson’s trichrome, positioned in the basal portion of the epithelium.

**Fig. 4:**
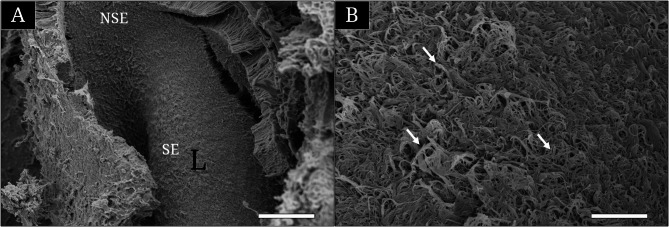
Scanning electron micrographs showing the lamella (**A**) within the olfactory chamber of Rhinogobius brunneus and its surface structure (**B**). White arrows indicate motile cilia. NSE, non-sensory epithelium; SE, sensory epithelium. Bars indicate 50 μm in **A** and 5 μm in **B**

On scanning electron microscopy, a longitudinal lamella was observed within the olfactory chamber (OC) protruding from its basal region (Fig. 4 A). The lamella was covered with numerous cilia, which were embedded in a layer of slimy mucus (Fig. 4 B).

## Discussion

The amur goby *R. brunneus* inhabiting Jeonju-cheon stream exhibits consistent anatomical and histological characteristics of the olfactory organ, regardless of seasonal differences in stream flow between June (low discharge) and July (high discharge). These characteristics were as follows: (i) a short tubular nostril, (ii) a single longitudinal lamella, and (iii) two accessory sacs (ethmoidal and lacrimal sacs; and iv) SE consisting of ORNs, SCs, LCs and BCs, and v) NSE with SECs, BCs, and MCs. Moreover, on scanning electron microscopy, there was an abundance of cilia in the SE, which may enhance its chemosensory efficiency under fluctuating hydrological conditions. These findings are similar to the cellular composition described in previous studies of the olfactory systems of other Gobiidae species that prefer to lentic habitat with stagnant flow and shallow depths (Belanger et al. 2003; Ghosh 2020).

The tubular AN generally works in conjunction with accessory nasal sacs for water ventilation into the OC, and in stagnant aquatic environments, this tubular structure facilitates water inflow through the pumping action of the nasal sacs induced by cranial oral bone movement, thereby enhancing suction force (Atta 2013). Variation in the number of accessory nasal sacs has been identified as a structural trait associated with the olfactory activity of gobies adapted to stagnant water or sedentary ecological lifestyles (Kuciel 2013; Kim et al. 2019). Therefore, such nostril and accessory nasal sacs (ethmoidal and lacrimal sacs) of *R*. *brunneus* represent morphological adaptions that promote water circulation into the OC, enabling sustained olfactory function in environments with reduced flow, stagnant water, or sedentary habits on rock and sand bottoms.

The single longitudinal lamella pattern of *R*. *brunneus* has also been observed in other small gobies: the round goby *Neogobius melanostomus* (Belanger et al. 2003), the freshwater tank goby *Glossogobius giuris* (Ghosh 2020), and the gluttonous goby *Chaenogobius gulosus* (Kim and Park 2021). It indicates a relatively narrow olfactory epithelial area and a simplified structure, in contrast to the multiple lamellae observed in migratory or pelagic species that rely heavily on olfaction in more dynamic or variable environments (Kasumyan 2004). When evaluating the olfactory sensitivity of teleost fishes, it is essential to consider the total surface area of the sensory epithelium (Adair et al. 2018). Nevertheless, a higher number of lamellae generally augments the SE surface area within the spatial constraints of the nasal cavity, thereby contributing to elevated abundance of ORNs (Kajiura et al. 2005). Thus, *R*. *brunneus* with a single lamella possess relatively low sensitivity, suggesting it is more reliant on vision than on olfaction for perceiving the surrounding environment and fulfilling ecological behaviors, thus falling among the “eye fish” group among the three categories of fishes (nose-fish, eye-nose fish, eye fish) proposed by Teichmann (1962). In addition, single longitudinal lamellae compared with other Gobiidae fishes are considered a taxonomic characteristic in its different number and morphology at least in Gobiiforms (Ma and Wang 2010; Ghosh 2020).

The MCs observed in the olfactory epithelium of *R*. *brunneus* were goblet shaped, resembling a grape cluster, and contained conspicuous mucous granules that stained bright red on H&E and deep red on Masson’s trichrome staining. However, in the skin of *R*. *brunneus*, mucous cells exhibited a faint and colorless reaction to H&E as well as Masson’s trichrome staining, showing a contrasting result to the mucous cells in the olfactory organ (Kim et al. 2022). The MCs of the olfactory organ in other gobiid fishes have exhibited variable color and structure on H&E and Masson’s trichrome as follows: (i) slimy mucin with light violet and dark red color in *Luciogobius guttatus* (Kim and Park 2016) and (ii) slimy mucin with a faint reaction in *Glossogobius giuris* (Ghosh 2020) and *Synechogobius hasta* (Kim 2024), and (iii) slimy mucin with pink color in *Periophthalums modestus* (Kim et al. 2019) and *Boleophthalmus pectinirostris* (Kim and Park 2020). Based on previous reports, mucous cells of *R*. *brunneus* tend to resemble those of Gobiidae species that inhabit terrestrial-like environments, hypoxic layers, or drought-prone conditions. In particular, the mucous granules of *R*. *brunneus* stained intensely red on both eosin and Masson’s trichrome, due to the affinity of eosin for the positively charged groups of basic amino acids and the affinity of acid fuchsin and Biebrich scarlet for proteinaceous cytoplasm (Park and Kim 2001; Sarasquete 2005). This can be interpreted as evidence that mucins or glycoproteins are abundant components of the mucus. Such mucous contents are generally reported to function as a physical barrier that protects the SE from suspended particles, potential toxic substances, and desiccation (Niklasson 2013). Accordingly, mucous granules in *R*. *brunneus* might play a crucial role in maintaining and enhancing olfactory function under conditions of low water flow or hypoxia.

Consequently, the olfactory organ of *R*. *brunneus* showed adaptation to stagnant aquatic environments and sedentary swimming behavior, and the proteinaceous mucus granules likely play a primary role in protecting the olfactory epithelium and cells under static and hypoxic conditions.

## Conclusions

This research investigated the anatomical and histological organization of the olfactory organ in the Korean amur goby *R. brunneus*, focusing on characters adapted to its benthic lifestyle and hypoxic, shallow, and stagnant stream habitat. Microscopy revealed the following features of the goby’s olfactory organ: (i) a short tubular nostril, (ii) a single longitudinal lamella, (iii) two accessory sacs (ethmoidal and lacrimal sacs), (iv) olfactory epithelium subdivided into sensory and non-sensory regions, and (v) proteinaceous granular mucous cells in the non-sensory epithelia. These findings suggest that *R*. *brunneus* is adapted for efficient olfactory activity in Korean streams characterized by low oxygen and stagnant, shallow water.

## Supplementary Information

Supplementary Material 1.

Supplementary Material 2.

## Data Availability

Not applicable.

## Abbreviations

AN: Anterior nostril
BC: Basal cell
ENS: Ethmoidal nasal sac
GA: Glutaraldehyde
H&E: Hematoxylin and eosin
L: Lamella
LC: Lymphatic cell
LNS: Lacrymal nasal sac
MC: Mucous cell
NSE: Non-sensory epithelia
OC: Olfactory chamber
ORN: Olfactory receptor neuron
PN: Sosterior nostril
SC: Supporting cell
SE: Sensory epithelia
SEC: Stratified epithelial cell
