# Supplementary material for: Anatomy and histology of the olfactory organ of Korean amur goby Rhinogobius brunneus (Gobiiformes, Gobiidae)
Source: Appl Microsc. 2025 Nov 29;55:12. doi: 10.1186/s42649-025-00116-4 (PMC12664872; doi:10.1186/s42649-025-00116-4)
Supplement: Supplementary file 1 — Supplementary Material 1. [file 42649_2025_116_MOESM1_ESM.docx]

Reviewer 1

Authors’ report

**Thank you very much for your nice comments on our manuscript and we tried to revise as much as possible based on your points. We are sure that your review makes our paper available and eligible to submit Applied Microscopy. Once more, we appreciate your cooperation. We indicate all the revised texts in red in the main text.**

This research paper is suitable for Applied Microscopy. But, some minor revision is needed.

In page 4, lane 61 : the Korean peninsula -> the Korean Peninsula

Answer: We revised.

In page 5, lane 99 : The is no model name of light microscope.

Answer: We revised.

In page 6, anatomy : There is no description of Fig. 2B including ENS, LNS and L.

Answer: The ENS and LNS are described in results as follows: “The ENS lies below the posterior nostril and extends medially between orbits, whereas the LNS is positioned in the posterior region of the OC and ventrally into the suborbital region.”. And L is “a single longitudinally-folded lamella”.

In page 6, histology : There is no description of Fig. 3B including LC, ORN, BC, and SC. or (Fig. 4B)? Isn't it 3B?

Answer: It was written in the results as follows. “ORNs were bipolar neurons characterized by an elongated nucleus and cytoplasm extending from the basement membrane to the epithelial surface. On H&E staining, their nuclei appeared purple, whereas the cytoplasm was faintly pink. SCs were cylindrical cells extending from the basal layer to the surface, each containing an oval nucleus weakly stained in purple and broad cytoplasm under H&E. BCs were rounded cells located just above the basement membrane, distinguished by a darkly stained purple nucleus. LCs were the smallest circular cells, intensely violet in color, and distributed mainly in the basal and upper epithelial layers.

In page 10, Abbreviations : There is no LC: lymphatic cell. Also check the ENS, LNS, and L.
Answer: We revised.

There is no figure legends including scale bar and abbreviations.

Answer: The figure legends, with scale bars and abbreviations, were separated from the main manuscript and added as supplementary materials file.
