# Supplementary material for: Anatomy and histology of the olfactory organ of Korean amur goby Rhinogobius brunneus (Gobiiformes, Gobiidae)
Source: Appl Microsc. 2025 Nov 29;55:12. doi: 10.1186/s42649-025-00116-4 (PMC12664872; doi:10.1186/s42649-025-00116-4)
Supplement: Supplementary file 2 — Supplementary Material 2. [file 42649_2025_116_MOESM2_ESM.docx]

Reviewer 2

Authors’ report

This manuscript presents a comprehensive anatomical and histological investigation of the olfactory organs in the brown goby (Rhinogobius brunneus), a species inhabiting low-oxygen river environments in Korea. The authors employ both optical and electron microscopy to enhance the reliability of their observations, and the ecological framing of the discussion is well-integrated and appropriate. The study offers valuable insights and is deemed suitable for publication following revisions.

**Answer: Thank you very much for your nice comments on our manuscript and we tried to revise as much as possible based on your points. We are sure that your review makes our paper available and eligible to submit Applied Microscopy. Once more, we appreciate your cooperation.**

Recommended Revisions:
1. Abbreviation Consistency
- The manuscript inconsistently uses singular and plural forms of terms (e.g., "olfactory epithelium" vs. "olfactory epithelia"). A thorough review is needed to ensure uniform usage.
- Abbreviations are defined in plural form but used inconsistently throughout the text. It is recommended to define abbreviations in singular form (e.g., "NSE: non-sensory epithelium; SE: sensory epithelium") and use plural forms like "NSEs" in the body text where appropriate.

**Answer: We revised.**

2. Unit Formatting
- The unit "µm" is incorrectly written as "um" in several instances. This should be corrected to maintain scientific accuracy.

**Answer: We revised.**

3. Reference Formatting
- Some references do not conform to the formatting guidelines of Applied Microscopy. All references should be revised accordingly.
- Example correction:
H.T. Kim, J.Y. Park, The anatomy and histoarchitecture of the olfactory organ in the Korean flat-headed goby Luciogobius guttatus (Pisces; Gobiidae). Appl Microsc 46, 51-57 (2016)
-> HT Kim, JY Park, The anatomy and histoarchitecture of the olfactory organ in the Korean flat-headed goby Luciogobius guttatus (Pisces, Gobiidae). Appl. Microsc. 46, 51-57 (2016).

**Answer: We revised.**

4. Figure Legends and Labels
- Figure 2: Scale bar is missing and should be added.
- Figure 3: Cell types need clearer labeling. For instance, "MC" is mentioned but not shown, and the arrowhead lacks explanation.
- Figure 4: More detailed labeling is needed for the surface structures of the lamellae (e.g., cilia, microvilli, epithelial junctions). Even the areas corresponding to the SE and NSE are unclear.

**Answer: We revised.**
